# Association of Human Leucocyte Antigen Polymorphism with Coronavirus Disease 19 in Renal Transplant Recipients

**DOI:** 10.3390/vaccines10111840

**Published:** 2022-10-30

**Authors:** Narayan Prasad, Brijesh Yadav, Swayam Prakash, Deependra Yadav, Ankita Singh, Sonam Gautam, Dharmendra Bhadauria, Anupama Kaul, Manas Ranjan Patel, Manas Ranjan Behera, Ravi Shankar Kushwaha, Monika Yachha

**Affiliations:** Department of Nephrology and Renal Transplantation, Sanjay Gandhi Postgraduate Institute of Medical Sciences, Lucknow 226014, India

**Keywords:** human leucocyte antigen, anti-SARS-CoV-2 antibody, seroconversion, alleles

## Abstract

Human leucocyte antigens (HLAs) are highly polymorphic glycoproteins expressed at the surface of all nucleated cells. It is required for the SARS-CoV-2 peptide antigen presentation to immune cells for their effector response. However, polymorphism in HLA significantly impacts the binding of SARS-CoV-2 antigenic peptide to the HLA pocket and regulates immune activation. In this study, 514 renal transplant recipients (RTRs) were recruited from the outpatient department and categorized either into symptomatic (*n* = 173) or asymptomatic groups (*n* = 341) based on Coronavirus disease-19 (COVID-19) symptoms. The anti-SARS-CoV-2 spike protein-specific IgG antibody titer was measured by chemiluminescent microparticle immune-assay methods in 310 RTRs. The HLA details of 514 patients were retrieved from the electronic medical records and analyzed retrospectively. We found that HLA antigen allele A*24 was significantly associated with asymptomatic infection in 22.78%, HLA C*02 in 4.51%, DRB1*12 in 10.85%, and HLA DQA1*02 in 27.74% of RTRs. Whereas HLA A*29 in 3.46%, A*33 in 26.01%, B*13 in 10.40%, DRB1*10 in 4.62%, DRB1*15 in 39.30%, DRB1*30 in 1.15%, and DQA1*60 in 3.57% of RTRs were associated with symptomatic infection. HLA DRB1*13 and DRB1*15 were associated with moderate to severe degrees of COVID-19 disease. The seroconversion rate in asymptomatic patients was 118/137 (86.13%), had a median titer of 647.80 au/mL, compared to symptomatic patients 148/173 (85.54%) with a median titer of 400.00 au/mL, which was not significant between the two groups (*p* = 0.88 and 0.13). In conclusion, HLA alleles A*24, C*02, DRB1*12, and DQA1*02 were significantly associated with asymptomatic infection, and A*29, A*33, B*13, DRB1*10, DRB*15, and DRB1*30 were significantly associated with symptomatic infection. HLA DRB1*13 and DRB1*15 were associated with moderate to severe degrees of COVID-19 disease.

## 1. Introduction

Severe acute respiratory syndrome corona virus-2 (SARS-CoV-2) infection emerged as a pandemic in December 2019 from Wuhan, China. Until 12 August 2022, 585 million people tested positive for SARS-CoV-2, and 6.4 million people succumbed to death [1]. Several co-morbid factors, such as age, obesity, diabetes, hypertension, and human leucocyte antigen (HLA) polymorphism, have been reported to be associated with infection and patient outcomes [2]. However, people from different ethnicities responded differently to the SARS-CoV-2 pandemic [3]. Many people develop mild to severe COVID-19 symptoms while others remain asymptomatic, suggesting various degrees of immune activation against the SARS-CoV-2 virus. The immune system of RTRs remains compromised due to the maintenance of immunosuppression. They are vulnerable to higher SARS-CoV-2 infection, mortality, and poor seroconversion rate than the general population [4,5]. 

For immune cell activation, a presentation of virus antigen through the HLA to T and B cells is required. There are more than 27,000 alleles in HLA, making an individual distinct from others. HLAs are encoded by the gene located at the short arm of chromosome 6p21.3. A polymorphism in HLA is associated with different cellular and humoral immune responses against antigens and vaccines [6]. HLA class 1 comprises A, B, and C, whereas 2 comprises DP, DQ, and DR. The polymorphism of these two classes of HLA is associated with different susceptibility to developing autoimmune diseases and infection clearance [7,8,9].

HLAs are unique glycoproteins expressed over the cell surface of all nucleated cells. HLAs are adopted for presenting pathogenic antigens to immune cells to trigger the immune response. Antigens from the microbial components and self-altered cells are endocytosed by the antigen-presenting cell (APCs), such as macrophages, Dendritic, B cells, and other nucleated cells. After intracellular processing, antigen peptides are presented through HLA to cognate T and B cell receptors for triggering appropriate cellular responses [10]. A huge polymorphism occurs in the nucleotide sequence of the gene encoding for HLA peptides [11]. The antigen peptide epitope binds to the HLA pocket peptide through a non-covalent bond; thus, the nature of the amino acid in the HLA binding pocket may affect the binding affinity with antigen epitopes, resulting in the activation of different degrees of the cellular and humoral immune response [10]. 

HLA polymorphisms are linked with the different susceptibility of microbial infections such as influenza-A (H1N1) [12], hepatitis B virus (HBV) [13], hepatitis C virus (HCV) [14], dengue [15], HIV [16], and tuberculosis [9]. Different populations and races responded to the SARS-CoV-2 virus differentially [3]. Even in India, people from other states reacted to the virus differently [17]. It suggests an association of HLA polymorphism in differential susceptibility for acquiring SARS-CoV-2 infection and immune response. However, there is a paucity of data on the association of HLA polymorphism with the severity of COVID-19 disease on immunocompromised renal transplant recipients (RTRs). Therefore, in the current study, we have retrospectively analyzed the HLA alleles polymorphism association with symptomatic and asymptomatic SARS-CoV-2 infection and COVID-19 severity in RTRs.

## 2. Material and Methods

### 2.1. Patient Recruitment

A total of 514 live-related renal transplant recipients were recruited from the outpatient department, visiting for routine follow-ups. All symptomatic patients had reverse transcriptase polymerase chain reaction (RT-PCR) positive SARS-CoV-2 infection. Patients and family members were interviewed about the SARS-CoV-2 symptoms. Based on self-reported symptoms of COVID-19 disease, such as myalgia, fever, dry cough, loss of taste and smell, and tiredness, patients were categorized as either asymptomatic (*n* = 314) with no COVID-19 symptoms or symptomatic (*n* = 173), having any of the above symptoms. Based on the criteria laid down by the Revised Guidelines on Clinical Management of COVID-19, Ministry of Health and Family Welfare, the Government of India [18], COVID-19 was classified as mild when symptoms were present without features of viral pneumonia on imaging (X-ray chest or high-resolution computed tomography (HRCT) scans), moderate if manifestation were present, while severe disease refers to the presence of hypoxia with respiratory rate >30 breaths/min, severe respiratory distress, or SpO2 < 90% on room air, including acute respiratory distress syndrome (ARDS). 

### 2.2. Blood Sample Collection and Anti-Spike Protein Analysis

Anti-Spike protein IgG titer was analyzed as described in our previous study [19]. A 5-mL blood sample was collected in EDTA vials from the 310 patients after collecting written consent from each patient. The blood sample was centrifuged at 1500 rpm for plasma separation. Plasma was stored at −20 °C till the immunoglobulin-G (IgG) titer measurement. In brief, plasma was incubated with anti-SARS-CoV-2 antigen-coated paramagnetic beads and washed to remove nonspecific bindings. Following washing, an acridinium labeled anti-human IgG conjugate was added and incubated. The nonspecific binding of IgG was removed by washing, and a pre-trigger and trigger solution of hydrogen peroxide and sodium hydroxide was added; this resulted in a chemiluminescent mixture formation. The intensity of the chemiluminescent mixture was measured on the Architect platform (Abbott diagnostic, Longford, Ireland) in a relative light unit (RLU). Further samples of RLU were normalized with calibrators as per the World Health Organization’s guidelines [20,21].

### 2.3. Human Leucocyte Antigen Analysis

We have retrospectively analyzed the HLA alleles profile of 514 live-related renal transplant recipients from their electronic medical records for HLA-A, B DR, and in 239 RTRs for HLA-C and DQA1. These antigen alleles were determined previously at the time of transplantation by the polymerase chain reaction-single strand oligonucleotide (PCR-SSO) methods using LIFE CODES HLA Typing Kits (Immucor, Diagnostic, Springfield, IL, USA) on Luminex Xponent platform (version 3.1). All samples were typed at the alleles group level only.

### 2.4. Statistical Analysis

Continuous variables were expressed as mean ± standard deviation. Student’s *t*-test was used to compare the variables between the groups. Categorical variables were expressed in percentages. The antibody titer was expressed as the median and interquartile range (IQR). Fisher’s exact test or Chi-square test was used to compare the categorical values between the groups. A *p*-value < 0.05 was considered to be significant. Logistic regression was performed to determine the other predictors for symptomatic and asymptomatic infection. Statistical analysis was performed with the SPSS software version 20 (IBM, corporation, Armonk, NY, USA).

## 3. Result

### 3.1. Demographic and Clinical Characteristics of Patients

The demographic and clinical characteristics of the patients are shown in Table 1. There was a significant difference in the patient’s age, BMI, TLCs, eGFR, and induction regimen used between symptomatic and asymptomatic RTR.

### 3.2. HLA Class 1 Alleles and Association with COVID-19 Symptoms

We have found that HLA antigen alleles A*24 were significantly associated with asymptomatic infection in 22.78% of RTRs, whereas A*29 in 3.46% and A*33 in 26.01% of patients were significantly associated with symptomatic infection. Other HLA-A alleles, such as A*11 in 22.87% and A*68 in 10.55% of patients, were associated with asymptomatic infection. However, there was no significant difference between the symptomatic and asymptomatic COVID-19 RTRs.

Similarly, HLA B*13 alleles were significantly associated with symptomatic infection in 10.40% of RTRs. Similarly, other major alleles, such as HLA B*07 were associated with asymptomatic infection in 11.73% and B*35 in 20.52% of RTRs, and HLA B*40 was associated with symptomatic infection in 13.87% of RTRs. However, the difference was non-significant between the symptomatic and asymptomatic groups (Table 2). HLA C*02 allele was significantly associated with asymptomatic infection in 4.51% of RTRs. HLA C*03 in 22.61%, C*04 in 19.04%, C*07 in 26.19%, and C*12 in 8.33% of RTRs were associated with symptomatic infection, and HLA C*06 was associated with asymptomatic infection in 18.70% of RTRs. However, there was no significant difference between symptomatic and asymptomatic infection (Table 2).

### 3.3. HLA Class 2 Alleles and Association with COVID-19 Symptoms

We have analyzed the HLA class 2 antigen alleles’ association with COVID-19 disease severity. HLA DRB1*10 in 4.62%, DRB*15 in 39.30%, and DRB1*30 in 1.15% of RTRs were significantly associated with symptomatic SARS-CoV-2 infection. DRB1*12 was associated with asymptomatic infection in 10.85% of RTRs. Similarly, DRB1*11 in 9.67% and DRB1*14 in 10.26% of patients were associated with asymptomatic infection (Table 3).

Further, HLA DQA1*02 was significantly associated with asymptomatic infection in 27.74% of RTRs, whereas DQA1*60 was associated with symptomatic infection in 3.57% of RTRs (Table 3).

### 3.4. Anti-Spike Protein IgG Antibody Titer

We have compared the seroconversion rate and antibody titer in 310 RTRs with asymptomatic (*n* = 137) and symptomatic (*n* = 173) infections. The seroconversion rate in asymptomatic RTRs was 118/137 (86.13%) with a median titer of 647.80 au/mL compared to symptomatic RTRs 148/173 (85.54%), with a median titer of 400.00 au/mL, which was similar between the two groups (*p* = 0.88 and 0.13); as shown Table 4. Furthermore, RTRs with symptomatic infection have been home-quarantined in 137/173 (79.2%) and hospitalized in 36/173 (20.80%) patients. Patients with hospitalization had a higher seroconversion rate of 35/36 (97.2%) as compared to home quarantine 113/137 (82.48%) (*p* = 0.03) (Table 4). 

### 3.5. Association of HLA with the Severity of COVID-19

Further, we analyzed the severity of HLA class I and II alleles with COVID-19 symptoms severity in hospitalized RTRs (*n* = 36). We found only DRB1 alleles were significantly associated with COVID-19 symptoms severity. DRB1*13 alleles were associated with moderate severity of COVID-19 in 57.14% of patients. DRB1*15 was associated with a mild to moderate degree of COVID-19 severity in 42.5% and a severe degree in 33.3% of RTRs. While DRB1*16 and DRB1*40 were associated with severe COVID-19 in 33.3% of RTRs (Table 5).

### 3.6. Other Predictors of Asymptomatic and Symptomatic Infection

Patient’s age (OR = 0.98, 95% CI 0.95–0.99, *p* = 0.01), hemoglobin (OR = 0.96, 95%CI 0.85–1.09, *p* = 0.45), and eGFR (OR = 0.98, 95%CI 0.98–1.00, *p* = 0.02) were associated with asymptomatic infection, whereas total leucocyte count (OR = 1.14, 95%CI 1.04–1.25, *p* < 0.001) and BMI (OR = 1.03, 95% CI 0.98–1.08; *p* = 0.36) were associated with the symptomatic infection.

## 4. Discussion

In this study, we have found that human leucocyte antigen alleles A*24 in 22.87%, HLAC*02 in 4.52%, and HLA DQA1*02 in 27.74% of RTRs were significantly associated with asymptomatic infection, whereas HLA alleles HLA B*13 in 4.62%, DRB1*10 in 4.62%, DRB*15 in 39.30%, DRB1*30 in 1.15%, and DQA1*60 in 3.57% of patients were associated with symptomatic infection. The seroconversion rate and median titer of the antibody were similar between groups. HLA DRB1*13 and DRB1*15 were associated with a moderate to severe degree of COVID-19. 

Antibody formation is required to protect from the SARS-CoV-2 infection, which requires a cellular immune response to be reckoned after infection or vaccination. The SARS-CoV-2 virus binds to the angiotensin-converting enzyme receptor. It enters the cell for its multiplication [22], which is sensed by the intracellular recognition system and flagged through HLA on its surface as an alarming signal. After intracellular processing of the SARS-CoV-2 peptide, antigen-presenting cells present to T and B cells for helper, effector, and humoral responses. 

The antigen binding affinity to the HLA binding pocket primarily determines the potency of immune cell activation [23,24]. The HLA pocket is composed of the sequence of amino acids, which is encoded by the HLA genes. Due to microbial antigen overload, HLA genes undergo continuous shuffling of the nucleotide sequence resulting in huge polymorphism in HLA loci [25,26]. This results in a change in the nature of amino acids in the binding pocket, resulting in the overall strength of immune cell activation [11,23,27]. Both in vivo and in silico simulation shows that HLA-B*46:01 and HLA-B*54:01 in the Taiwanese population were associated with severe COVID-19, and HLA-B*1301 shows a protective association. In contrast, on subgroup analysis, we observed that HLA-B*13 was associated with a mild to moderate degree of symptomatic disease and 100% seroconversion rate with median anti-Spike IgG titer 1123.00 au/mL, which suggests robust seroconversion and efficient clearance of the virus. However, an in-silico study shows a weak antigen-presenting capacity of HLA-B*46:01 [24].

Further, one Italian study has shown an association of HLA-DRB1*15:01, DQB1*06:02, and B*27:07 with COVID-19 severity [28]. However, we observed only HLA-DRB1*15 association with symptomatic infection in 39.30% of RTRs, an in silico study has shown that HLA-DRB1*15 alleles can bind with different variants of SARS-CoV-2 peptides with multiple binding sites and present it to T and B cells with the highest capacity and generate a potent immune response, which is associated with a humoral immune response as seroconversion. This suggests exaggerated immune activation may be the cause of COVID-19 severity. However, this helps in the early clearance of the virus. We observed that HLA-DRB1*15 was significantly associated with symptomatic infection, and HLA-DRB1*13 and 15 were associated with mild to severe degrees of COVID-19 disease, which suggests both alleles may link with a robust immune response and seroconversion. In symptomatic groups, seroconversion was observed in 85.54% of patients. Since RTRs remain on continuous maintenance of triple immunosuppressive regimens, such as Tacrolimus, Mycophenolate mofetil, and Prednisolone, Mycophenolate is reported to hamper seroconversion against SARS-CoV-2 peptide significantly [27]. Thus, in these RTRs, overactivation did not happen, 79.2% of patients needed home quarantine, and 20.80% were hospitalized in the symptomatic group with mild to moderate COVID-19 in the majority. In the Mexican population, HLA-DRB1*01 was negatively associated with COVID-19-associated fatality [29]. However, in our current study, we did not observe any significant association of DRB1*01 with COVID-19 severity.

Further, in a normal Saudi Arabia population, HLA-A*01, B*56, DRB1*04, and DRB1*08 were significantly associated with COVID-19 infection, and HLA-A*03 and C*06 were associated with mortality. However, we did not observe any significant association with COVID-19 for these alleles. However, the frequency of HLA DRB1*04 was increased in symptomatic patients, and C*06 and DRB1*08 were higher in asymptomatic patients [30].

Due to immunosuppression in RTR, seroconversion is less likely to happen, as evidence emerged from the western RTRs [31,32]. In this study, we have observed that 60% of RTRs did not experience any symptoms of COVID-19 and had a seroconversion rate of 86.13%, with a median titer of 647.80 au/mL compared to 85.54% seroconversion and median titer of 400.0 au/mL in the symptomatic group, which is consistent with our previous study on the seroconversion rate after whole virus-based vaccination in RTRs [33,34]. The observations further suggest that immunosuppression does not significantly hamper the seroconversion rate and may help in the reduction of COVID-19 severity. A few other studies have also shown that immunosuppressive steroids have significantly reduced inflammation-associated mortality in COVID-19 [35,36]. However, it delays viral clearance. Although, it is critical to decide the ideal window period after which the immunosuppressives should be started to prevent an exaggerated immune response. Our previous study showed that the whole virus antigens are more efficient in triggering seroconversion and clearance of the virus in RTRs [33]. The present study’s findings suggest that asymptomatic COVID-19 may be due to effective and rapid clearance of the virus. COVID-19 severity may be due to either over/under activation of an immune response. Patients with severe COVID-19 often show the features of activated helper Th17 and CD8^+^cytotoxic-T cells, inflammatory cytokines, and lymphopenia [37,38]. The activation of T-helper and Cytotoxic T-cells may have a detrimental effect on the allograft in itself [39,40,41]. We did not observe any significant deterioration in allograft function in this study. 

The limitation of our study is that HLA genotyping for HLA-C and DQA1 was available for only 239 patients, and the anti-SARS-CoV-2 spike protein antibody titer was measured in only 310 RTRs. The majority of RTRs included in the study were male (87.15%), as this was the proportion of patients going for transplantation in India. The small sample size remains the major limitation. Further, to establish the HLA association with COVID-19 disease severity, a larger study would be required. 

## 5. Conclusions

HLA alleles A*24, C*02, DRB1*12, and DQA1*02 were significantly associated with asymptomatic COVID-19 and A*29, A*33, B*13, DRB1*10, DRB*15, and DRB1*30 were significantly associated with symptomatic COVID-19. HLA DRB1*13 and DRB1*15 were associated with moderate to severe degrees of COVID-19. The seroconversion rate was 85.54% in symptomatic and 86.13% in asymptomatic COVID-19 patients. The median antibody titer in symptomatic was 400 au/mL and in asymptomatic patients was 647.80 au/mL. 

## Figures and Tables

**Table 1:** Demographic and clinical profile of RTRs with asymptomatic and symptomatic infection.

Characteristics		Asymptomatic (*n* = 341)	Symptomatic (*n* = 173)	*p*-Value
**Patients Age (Years)**		42.53 ± 11.35	39.76 ± 11.64	0.01
Gender	M (%)	300 (87.97)	148 (85.54)	0.48
	F (%)	41 (12.02)	25 (14.45)	
Post-transplant interval (months)		84.92 ± 65.58	86.41 ± 65.14	0.80
BMI (kg/m^2^)		23.20 ± 5.22	24.56 ± 5.19	0.01
Hemoglobin (mg/dL)		12.74 ± 1.81	12.74 ± 1.94	0.99
TLC (×10^3^/µL)		7.14 ± 2.44	7.79 ± 2.59	0.005
Baseline serum creatinine (mg/dL)		0.89 ± 0.37	0.83 ± 0.34	0.10
Serum creatinine (mg/dL)		1.57 ± 0.78	1.62 ± 0.48	0.44
BUN (mg/dL)		23.20 ± 9.58	24.29 ± 12.66	0.27
Tacrolimus level (µg/L)		5.55 ± 2.03	5.91 ± 2.73	0.09
eGFR (mL/min/1.73 m²)		64.96 ± 31.75	58.98 ± 21.37	0.026
Induction	Basiliximab	78	57	0.046
	ATG	85	35	
	No Induction	178	81	
Patient blood group	A^+ve^	79	44	0.76
	B^+ve^	142	64	
	AB^+ve^	34	17	
	O^+ve^	86	48	
Immunosuppression	Tacrolimus	320	169	0.055
	Cyclosporin	21	4	

BMI—Body mass index, TLCs—Total leucocyte count, BUN—Blood urea nitrogen, eGFR—Estimated glomerular filtration rate, ATG—Anti-thymocyte-globulin.

**Table 2:** HLA-A alleles frequency in RTRs with asymptomatic and symptomatic infection.

**HLA Alleles**	**Asymptomatic *n* = 341 (%)**	**Symptomatic *n* = 173 (%)**	***p*-Value**
A*01	7 (2.052)	3 (1.73)	0.8
A*02	12 (3.51)	10 (5.78)	0.22
A*03	22 (6.45)	10 (5.78)	0.76
A*09	1 (0.29)	0 (0)	0.47
A*11	78 (22.87)	23 (13.29)	0.09
A*13	1 (0.29)	0 (0)	0.47
A*20	1 (0.29)	0 (0)	0.47
A*23	1 (0.29)	0 (0)	0.47
A*24	78 (22.87)	26 (15.02)	0.03
A*25	10 (2.93)	2 (1.15)	0.2
A*26	17 (4.98)	10 (5.78)	0.7
A*28	1 (0.29)	0 (0)	0.47
A*29	2 (0.58)	6 (3.46)	0.01
A*30	2 (0.58)	3 (1.73)	0.2
A*31	4 (1.173)	5 (2.89)	0.16
A*32	7 (2.05)	6 (3.46)	0.33
A*33	57 (16.71)	45 (26.01)	0.01
A*34	1 (0.29)	0 (0)	0.47
A*36	0 (0)	1 (0.57)	0.16
A*38	0 (0)	1 (0.57)	0.16
A*66	2 (0.58)	0 (0)	0.31
A*68	36 (10.55)	21 (12.13)	0.59
A*74	1 (0.29)	0 (0)	0.47
A*80	0 (0)	1 (0.57)	0.16
B*04	9 (2.63)	7 (4.04)	0.38
B*05	3 (0.87)	0 (0)	0.21
B*07	40 (11.73)	8 (4.62)	0.09
B*08	8 (2.34)	5 (2.89)	0.7
B*12	1 (0.29)	0 (0)	0.47
B*13	10 (2.93)	18 (10.40)	<0.0001
B*14	3 (0.87)	0 (0)	0.21
B*15	42 (12.31)	23 (13.29)	0.75
B*18	9 (2.63)	6 (3.46)	0.59
B*27	16 (4.69)	6 (3.46)	0.51
B*33	1 (0.29)	0 (0)	0.47
B*35	70 (20.52)	32 (18.49)	0.58
B*37	13 (3.81)	8 (4.62)	0.66
B*38	4 (1.17)	3 (1.73)	0.6
B*39	7 (2.052)	1 (0.57)	0.2
B*40	33 (9.67)	24 (13.87)	0.15
B*41	2 (0.58)	1 (0.57)	0.98
B*44	25 (7.33)	13 (7.51)	0.94
B*48	1 (0.29)	0 (0)	0.47
B*50	5 (1.46)	1 (0.57)	0.37
B*51	6 (1.75)	3 (1.73)	0.98
B*52	15 (4.39)	3 (1.73)	0.12
B*53	2 (0.58)	0 (0)	0.31
B*55	3 (0.87)	2 (1.15)	0.75
B*56	2 (0.58)	0 (0)	0.31
B*57	2 (0.58)	1 (0.57)	0.98
B*58	3 (0.87)	0 (0)	0.21
B*70	5 (1.46)	7 (4.04)	0.06
B*80	1 (0.29)	1 (0.57)	0.62
**HLA-Alleles**	**Asymptomatic *n* = 155 (%)**	**Symptomatic *n* = 84 (%)**	***p*-Value**
C*01	5 (3.22)	1 (1.19)	0.33
C*02	7 (4.51)	0 (0)	0.04
C*03	22 (14.19)	19 (22.61)	0.09
C*04	24 (15.48)	16 (19.04)	0.48
C*05	4 (2.58)	5 (5.95)	0.19
C*06	29 (18.70)	10 (11.90)	0.17
C*07	39 (25.16)	22 (26.19)	0.86
C*08	3 (1.93)	2 (2.38)	0.81
C*12	12 (7.74)	7 (8.33)	0.87
C*14	3 (1.93)	0 (0)	0.2
C*15	4 (2.58)	2 (2.38)	0.92
C*17	1 (0.64)	0 (0)	0.46
C*20	1 (0.64)	0 (0)	0.46
C*70	1 (0.64)	0 (0)	0.46

* indicates separation.

**Table 3:** HLA-DRB1 alleles frequency in RTRs with asymptomatic and symptomatic infection.

**HLA Alleles**	**Asymptomatic *n* = 341 (%)**	**Symptomatic *n* = 173 (%)**	***p*-Value**
DRB1*01	8 (2.34)	4 (2.31)	0.98
DRB1*03	4 (1.17)	0 (0)	0.15
DRB1*04	11 (3.22)	9 (5.20)	0.27
DRB1*07	24 (7.03)	14 (8.09)	0.66
DRB1*08	9 (2.63)	1 (0.57)	0.10
DRB1*10	3 (0.87)	8 (4.62)	0.005
DRB1*11	33 (9.67)	15 (8.67)	0.72
DRB1*12	37 (10.85)	7 (4.04)	0.014
DRB1*13	43 (12.60)	24 (13.87)	0.78
DRB1*14	35 (10.26)	14 (8.09)	0.43
DRB1*15	120 (35.19)	68 (39.30)	<0.001
DRB1*16	7 (2.05)	3 (1.73)	0.80
DRB1*30	0 (0)	2 (1.15)	0.047
DRB1*40	1 (0.29)	2 (1.15)	0.22
DRB1*70	4 (1.17)	2 (1.15)	0.98
DRB1*80	2 (0.58)	0 (0)	0.31
**HLA-Alleles**	**Asymptomatic *n* = 155 (%)**	**Symptomatic *n* = 84 (%)**	***p*-Value**
DQA1*02	43 (27.74)	12 (14.28)	0.018
DQA1*03	66 (42.58)	37 (44.04)	0.82
DQA1*04	3 (1.93)	3 (3.57)	0.43
DQA1*05	28 (18.06)	18 (21.42)	0.53
DQA1*06	14 (9.03)	8 (9.52)	0.9
DQA1*20	1 (0.64)	2 (2.38)	0.24
DQA1*50	0 (0)	1 (1.19)	0.17
DQA1*60	0 (0)	3 (3.57)	0.01

* indicates separation.

**Table 4:** Seroconversion rate in symptomatic (home quarantined or hospitalized) and asymptomatic RTRs.

		Asymptomatic (*n* = 137)	Symptomatic (*n* = 173)	*p*-Value	
Seroconversion	Yes (%)	118 (86.13%)	148 (85.54%)	0.88	
	No (%)	19 (13.86%)	25 (14.45%)		
Median Antibody titer (au/mL) (IQR)		647.80 (167.75–2524.25)	400.00 (154.10–1670.10)	0.13	
**Symptomatic Infection (*n* = 173)**					
	**HQ (*n* = 137, 79.2%)**		**HSP (*n* = 36, 20.80%)**		***p*-Value**
Seroconversion	Yes	No	Yes	No	
	113 (82.48%)	24 (17.51%)	35 (97.22%)	1 (2.77%)	0.030

HQ—Home quarantined, HSP—Hospitalized, IQR—Interquartile range.

**Table 5:** HLA association with severity of COVID-19 disease.

HLA	Mild (*n* = 26)	Moderate (*n* = 7)	Severe (*n* = 3)	*p*-Value
DRB1*07 (%)	1 (3.84)	0 (0)	0 (0)	0.005
DRB1*11 (%)	5 (19.23)	0 (0)	0 (0)	
DRB1*13 (%)	4 (15.38)	4 (57.14)	0 (0)	
DRB1*14 (%)	4 (15.38)	0 (0)	0 (0)	
DRB1*15 (%)	11 (42.30)	3 (42.85)	1 (33.3)	
DRB1*16 (%)	0 (0)	0 (0)	1 (33.3)	
DRB1*30 (%)	1 (3.84)	0 (0)	0 (0)	
DRB1*40 (%)	0 (0)	0 (0)	1 (33.3)	

* indicates separation.

## Data Availability

The data supporting this study’s findings are available from the corresponding author upon reasonable request.
